# Mechanisms and Efficacy of Chinese Herbal Medicines in Chronic Kidney Disease

**DOI:** 10.3389/fphar.2020.619201

**Published:** 2021-03-29

**Authors:** Mingming Zhao, Yi Yu, Rumeng Wang, Meiying Chang, Sijia Ma, Hua Qu, Yu Zhang

**Affiliations:** ^1^ Department of Nephrology, Xiyuan Hospital, China Academy of Chinese Medical Sciences, Beijing, China; ^2^ Beijing University of Chinese Medicine, Beijing, China; ^3^ Xiyuan Hospital, China Academy of Chinese Medical Sciences, Beijing, China; ^4^ NMPA Key Laboratory for Clinical Research and Evaluation of Traditional Chinese Medicine, Beijing, China; ^5^ National Clinical Research Center for Chinese Medicine Cardiology, Beijing, China

**Keywords:** chronic kidney disease, Chinese herbal medicines, anti-inflammatory, antioxidative, anti-apoptotic, autophagy-mediated, antifibrotic

## Abstract

As the current treatment of chronic kidney disease (CKD) is limited, it is necessary to seek more effective and safer treatment methods, such as Chinese herbal medicines (CHMs). In order to clarify the modern theoretical basis and molecular mechanisms of CHMs, we reviewed the knowledge based on publications in peer-reviewed English-language journals, focusing on the anti-inflammatory, antioxidative, anti-apoptotic, autophagy-mediated and antifibrotic effects of CHMs commonly used in kidney disease. We also discussed recently published clinical trials and meta-analyses in this field. Based on recent studies regarding the mechanisms of kidney disease *in vivo* and *in vitro*, CHMs have anti-inflammatory, antioxidative, anti-apoptotic, autophagy-mediated, and antifibrotic effects. Several well-designed randomized controlled trials (RCTs) and meta-analyses demonstrated that the use of CHMs as an adjuvant to conventional medicines may benefit patients with CKD. Unknown active ingredients, low quality and small sample sizes of some clinical trials, and the safety of CHMs have restricted the development of CHMs. CHMs is a potential method in the treatment of CKD. Further study on the mechanism and well-conducted RCTs are urgently needed to evaluate the efficacy and safety of CHMs.

## Introduction

Chronic kidney disease (CKD), characterized by a glomerular filtration rate (GFR) of less than 60 ml/min/1·73 m^2^ and/or markers of kidney damage, is an increasing public health issue because of its high prevalence and increased risk of end-stage renal disease (ESRD), cardiovascular disease, and premature death (Matsushita et al., 2010; Webster et al., 2017). It is estimated that the prevalence of CKD worldwide is 8–16%, of which 78% is concentrated in middle- and low-income countries (Jha et al., 2013; Mills et al., 2015). People with CKD are five to 10 times more likely to die prematurely than they are to progress to ESRD (Webster et al., 2017). This increased risk of death rises exponentially as kidney function worsens, and is largely attributable to death from cardiovascular disease (Tonelli et al., 2006; Thompson et al., 2015).

The management of patients with CKD is focused on early detection or prevention, treatment of the underlying cause to curb progression, and attention to secondary processes that contribute to ongoing nephron loss (Romagnani et al., 2017). Angiotensin-converting enzyme inhibitor (ACEI) or an angiotensin receptor blocker (ARB), together with optimal blood pressure (BP) control, remains the mainstay treatment for retarding the progression toward ESRD (Viazzi et al., 2016). Despite treatment with agents, such as an ACEI and ARB, many studies have shown that there is incomplete blockade of the renin-angiotensin cascade evidenced by persistent or increasing plasma aldosterone levels. This phenomenon is commonly referred to as “aldosterone escape,” and is thought to be one of the main contributors to CKD progression (Lu et al., 2010). The Supportive vs. Immunosuppressive Therapy for the Treatment of Progressive immunoglobulin A Nephropathy trial and Therapeutic Evaluation of Steriods in IgA Nephropathy Global trial suggested that corticosteroids may reduce proteinuria but carry a higher risk of adverse events (Barbour and Feehally, 2017). Therefore, it is necessary to seek more effective and safe treatment for CKD, such as Chinese herbal medicines (CHMs).

Traditional Chinese medicine (TCM) is based on the principles of the “concept of holism” and “treatment based on syndrome differentiation.” Clinical trials and experimental studies have shown that CHMs have a great beneficial effect on the reduction of proteinuria and improvement of renal function. Although most of the studies on the treatment of kidney diseases with CHMs are published in Chinese-language journals, peer-reviewed articles published in this field have also dramatically increased (Zhong et al., 2015). In order to clarify the modern theoretical basis and molecular mechanisms of CHMs, we reviewed the knowledge based on publications in peer-reviewed English-language journals, focusing on the anti-inflammatory, antioxidative, anti-apoptotic, autophagy-mediated, and antifibrotic effects of CHMs commonly used in kidney disease. We also discussed recently published clinical trials and meta-analyses in this field.

## Mechanisms of Chinese Herbal Medicines in Treating Kidney Diseases

### Anti-inflammatory and Antioxidative Effects of Chinese Herbal Medicines

Persistent, low-grade inflammation is now considered to be the main cause of pathophysiological processes in kidney diseases (Mihai et al., 2018). Systemic persistent inflammation is also considered to be a major factor in the uremic phenotype (such as cardiovascular disease, protein energy wasting, depression, osteoporosis, and frailty), as well as a predictor of cardiovascular and all-cause mortality (Cobo et al., 2018). Oxidative stress and inflammation interact with each other and play crucial roles in the development of CKD (Xu et al., 2015). The kidney is a highly metabolic organ, and the mitochondria are rich in oxidative reactions, which makes it vulnerable to oxidative stress. Among patients with advanced CKD, increased oxidative stress is associated with complications, such as hypertension, atherosclerosis, inflammation, and anemia (Daenen et al., 2019). Inhibition of inflammation and oxidation is an important means to promote the remission of kidney diseases, which may also be one of the important mechanisms of TCM in the treatment of kidney diseases. CHMs commonly used in the treatment of kidney diseases have anti-inflammatory and antioxidant effects.

The officinal part of *Astragalus mongholicus* Bunge is the root. The anti-inflammatory and antioxidative effects of *Astragalus mongholicus* Bunge and its extractions have been investigated in animal models of kidney disease including diabetic nephropathy (Gao et al., 2012; Du et al., 2018; Zhang et al., 2019f), acute kidney injury (Gui et al., 2013), and unilateral ureteral obstruction (UUO) (Zhou et al., 2017b). Calycosin (C_16_H_12_O_5_), as a major active component of *Astragalus mongholicus* Bunge, reduced the expression of tumor necrosis factor-α (TNF-α) and interleukin-1β (IL-1β) in the cultured mouse tubular epithelial cells, and alleviated kidney injury in diabetic kidneys of db/db mice during the progression of diabetic renal injury, as indicated by the reduction of histological injury and immunohistochemical of inflammatory cytokines (Zhang et al., 2019f). Astragaloside IV may potentially protect against renal fibrosis by reducing oxidative stress and inflammation via transforming growth factor-β1 (TGF-β1)/Smads signaling or the toll-like receptor 4 (TLR4)/nuclear factor kappa-B (NF-κB) signaling pathway (Gui et al., 2013; Zhou et al., 2017b; Du et al., 2018).

The officinal parts of *Salvia miltiorrhiza* Bunge are the root and rhizome. *Salvia miltiorrhiza* Bunge and its extractions have been shown to reduce proteinuria and attenuate kidney injury in several animal models of kidney disease, including 5/6th renal ablation/infarction (Lin et al., 2019a), renal ischemic reperfusion injury (Ma et al., 2017), diabetic nephropathy (Hou et al., 2017), and doxorubicin-induced nephropathy (Liu et al., 2011). These effects associated with anti-inflammatory and antioxidative mechanisms have been observed in conjunction with these observed beneficial effects. Magnesium lithospermate B improves renal function, fibrosis, and inflammation in rats with chronic renal failure, and these effects are probably related to the increase in renal blood flow, reduction of oxygen consumption, and attenuation of renal hypoxia in the remnant kidney (Lin et al., 2019a). Salvianolic acid A attenuated oxidative stress induced by advanced glycation end products (AGEs), and subsequently alleviated inflammation and restored the disturbed autophagy in glomerular endothelial cell and diabetic rats (Hou et al., 2017). In addition, salvianolic acid B can therapeutically alleviate oxidative stress and inflammatory process via the modulating phosphatidylinositol 3-kinase (PI3K)/protein kinase B (AKT) signaling pathway (Ma et al., 2017). Tanshinone IIA sodium sulfonate treatment not only improved doxorubicin-induced nephropathy, but also regulated the expression of several proteins related with the cytoskeleton, oxidative (Liu et al., 2011).

The main officinal part of *Tripterygium wilfordii* Hook. f. is the root. *Tripterygium wilfordii* Hook. f. and its extractions, tripterygium glycoside and triptolid, have been reported to attenuate proteinuria and podocyte injury in many animal models of kidney diseases by suppression of inflammatory factors (transforming growth factor- β1 [TGF-β1], interleukin-2, and interferon-γ) (Wan et al., 2010), macrophage infiltration (Ma et al., 2013), oxidative stress (lactate dehydrogenase, malondialdehyde [MDA], and super oxide dismutase [SOD]) (Wan et al., 2020). With prolongation of treatment time, the efficacy of triptolide increased, and the effect was better in the high-dose group than in the low-dose group (Gao et al., 2010).

The main officinal part of *Abelmoschus manihot* (L.) Medik. is the flower. *Abelmoschus manihot* (L.) Medik., which is made into Huangkui capsule, has potent anti-inflammatory and antioxidative effects. It alleviated renal tubular epithelial-to-mesenchymal transition (EMT) by inhibiting the nucleotide-binding domain and the leucine-rich, repeat-containing family, pyrin-containing 3 inflammasome, and TLR4/NF-κB signaling in a diabetic nephropathy model in rats (Han et al., 2019), as well as reduced endoplasmic reticulum stress and c-Jun NH2-terminal kinase activation, and subsequently reduced the expressions of inflammatory genes (Ge et al., 2016). In addition, *Abelmoschus manihot* (L.) Medik. has been observed to have protective effects against injury of renal tubular epithelial cells (HK-2) involved in the mechanism of inhibiting the nicotinamide adenine dinucleotide phosphate (NADPH) oxidase/reactive oxygen species (ROS)/extracellular signal-regulated kinase (ERK) pathway (Cai et al., 2017).

The officinal part of *Pueraria montana* (Lour.) Merr. is the root. This plant is used in the treatment of diabetic nephropathy. Puerarin inhibited AGE-induced inflammation in mouse mesangial cells (Kim et al., 2010). It also decreased renal tubular injury in UUO mice by inhibiting oxidative stress via MAPK signaling (Zhou et al., 2017a).

The medicinal part of *Rheum palmatum* L. is the root and rhizome. Rhein, as a main active component of *Rheum palmatum* L., could improve the symptoms of uric acid nephropathy by decreasing the production of proinflammatory cytokines, including IL-1β, prostaglandin E, TNF-α, and TGF-β1 (Meng et al., 2015; Hu et al., 2020). Rhein not only alleviates renal interstitial pathological damage and collagen fibrils, but also improves renal function through the silent mating-type information regulation two homolog-3 (SIRT3)/forkhead box O3α signaling pathway (Wu et al., 2020b).

The medicinal part of *Paeonia lactiflora* Pall. is the root. *Paeonia lactiflora* Pall. has been observed to prevent macrophage activation in type 2 diabetic nephropathy by inhibiting TLR2/4 signaling (Zhang et al., 2017b). Further research has shown that paeoniflorin can affect macrophages by inhibiting the expression of inducible nitric oxide synthase and the production of TNF-α, IL-1β, and monocyte chemoattractant protein-1 *in vitro* and *in vivo*, but it cannot directly inhibit the activation of macrophages (Shao et al., 2019). Total glucosides of paeony can prevent diabetic related renal injury by inhibiting oxidative stress injury in diabetic rats (Su et al., 2010).

The medicinal part of Panax notoginseng (Burkill) F.H.Chen is the root and rhizome. *Panax notoginseng* (Burkill) F.H.Chen and panax notoginseng saponins could protect kidney from diabetes via the mechanism of upregulating SIRT1, therefore activating antioxidant proteins and inhibiting inflammation by decreasing the inflammatory cytokines (Du et al., 2016). Notoginsenoside R1 is a promising drug for the prevention and treatment of renal insufficiency through inhibition of the production of inflammatory cytokines induced by ischemic reperfusion and inhibition of oxidative stress (Fan et al., 2020). *In vitro*, notoginsenoside R1 protects human renal proximal tubular epithelial cells from lipopolysaccharide (LPS)-induced inflammatory injury by upregulating miR-26a and inactivating the NF-κB pathway (Liu et al., 2019b).

The medicinal part of *Panax ginseng* C.A.Mey. is the root and rhizome. Ginsenoside Rg1 has no effect on cell migration and ROS activity, but they alleviate the ROS release and migration impairment induced by LPS. Ginsenoside Rg1 has the potent anti-inflammatory effect of protecting HK-2 cells against LPS-induced inflammation via activation of the PI3K/AKT pathway and suppression of the NF-κB pathway (Ni et al., 2017a). Ginsenoside Rg1 could also effectively alleviate aldosterone-induced oxidative stress in the kidney (Wang et al., 2015c).

### Anti-apoptotic Effect of Chinese Herbal Medicines

Apoptosis, which is considered to be an important mechanism of cell death, is related to the pathological processes of cisplatin-induced renal injury, ischemic kidney injury, and polycystic kidney disease (Wiegele et al., 1998; Cummings and Schnellmann, 2002; Tao et al., 2005). Previous studies have demonstrated that apoptosis is closely correlated with the development and progression of renal diseases, including ischemic kidney injury (Wei et al., 2013), ischemia-reperfusion induced renal injury (Xu et al., 2019a), cisplatin-induced renal injury (Yang et al., 2018), and diabetic nephropathy (Peng et al., 2015). Therefore, inhibition of apoptosis may be an important target for the treatment of kidney disease.

Astragaloside IV could attenuate both UUO and TGF-β1-induced apoptosis, as well as prevent HK-2 cell injury in a dose-dependent manner (Xu et al., 2014a). It could also improve the histopathological changes in the diabetic kidney by reducing the expression of the apoptosis-related proteins cleaved caspase-3, Bcl2-associated X (Bax)/B-cell lymphoma-2 (Bcl-2) ratio (Ju et al., 2019). Magnesium lithospermate B has been shown to have an anti-apoptotic effect on attenuating kidney injury in 5/6 renal ablation/infarction model rats with chronic renal failure (Wang et al., 2019b). Tripterygium glycoside and triptolide had protective effects in adriamycin-induced nephrotic syndrome in rats (Wang et al., 2020) and aminonucleoside-induced podocytes injury (Yang et al., 2019b) by inhibiting activation of apoptosis. Pretreatment with *Abelmoschus manihot* (L.) Medik. decreased urinary albumin excretion in rats with early-stage diabetic nephropathy, which might have been accomplished by preventing kidney injury and podocyte apoptosis (Zhou et al., 2012). Treatment with puerarin could ameliorate renal fibrosis by inhibiting epithelial cell apoptosis induced by oxidative stress through MAPK signaling (Song et al., 2016; Zhou et al., 2017a). In podocytes, puerarin could inhibit high glucose-induced apoptosis and restore the expressions of heme oxygenase 1 (HMOX-1) and SIRT1 (Li et al., 2020b). Rhein alleviated apoptosis of renal tubular cell and renal fibrosis in rats with UUO by suppressing the expression of signal transducer and activator of transcription 3 phosphorylation (Chen et al., 2019d). In an *in vitro* study, paeoniflorin reduced caspase-3 and Bax and increased Bcl-2, suggesting that the apoptosis of podocytes induced by adriamycin was reduced (Lu et al., 2017). Notoginsenoside has been shown to have an anti-apoptotic effect against polymyxin E-induced nephrotoxicity (Zhang et al., 2019e), cisplatin-induced nephrotoxicity (Liu et al., 2014), ischemia-reperfusion injury (Liu et al., 2010), LPS-induced inflammatory damage (Liu et al., 2019b), and diabetic nephropathy (Zhang et al., 2019a). Ginsenoside Rg1 could protect HK-2 cells against LPS-induced apoptosis by activation of the PI3K/AKT pathway and suppression of the NF-κB pathway (Ni et al., 2017a). *Panax ginseng* C.A.Mey. also could have an effect against cisplatin-induced nephrotoxicity (Qi et al., 2019) and high glucose-induced renal injury (Ha and Ha, 2019) via inhibition of apoptosis.

### Autophagy-Mediated Effect of Chinese Herbal Medicines

Autophagy is a process of the cell cycle that includes the self-degradation and reconstruction of damaged organelles and proteins (Wollert, 2019). In clinical studies, the activation and inhibition of autophagy are associated with acute kidney injury, CKDs, diabetic nephropathy, and polycystic kidney diseases (Lin et al., 2019b). Oxidative stress, inflammation, and mitochondrial dysfunction are important mechanisms of many kidney diseases, regulating the activation and inhibition of autophagy (Kimura et al., 2017; Kaushal et al., 2019; Fujimura et al., 2020).

Astragaloside IV has been shown to have a renoprotective effect on relieving renal fibrosis and renal function in diabetic mice, and effects on podocyte EMT by regulating the SIRT1-NF-κB pathway and autophagy (Wang et al., 2019c). Astragaloside IV also prevents the progression of diabetic nephropathy by AMP-activated protein kinase (AMPK) *α*-promoted autophagy (Guo et al., 2017). Salvianolic acid A restored the dysfunction of autophagy in diabetic rats and glomerular endothelial cell via the receptor for advanced glycation end-products (RAGE)-NADPH-oxidase 4 axis (Hou et al., 2017). Tripterygium glycoside has a protective effect against podocyte injury induced by high glucose and puromycin aminonucleoside, and this effect is mediated by the activation of autophagy (Gong et al., 2018; Zhan et al., 2019). *In vivo* and vitro, puerarin protected podocytes from diabetes-induced injury through upregulated autophagy mediated by HMOX-1 and SIRT1 (Li et al., 2020b), as well as alleviated cadmium-induced cytotoxicity in primary rat proximal tubular cells by restoring autophagy, blocking lysosomal membrane permeabilization, and inhibiting the nuclear factor erythroid-2 related factor 2 pathway, which is intimately related with its antioxidant activity (Wang et al., 2019a). Autophagy activation accompanied with renal fibrosis in rats with adenine-induced renal tubular injury, and both autophagy and renal fibrosis could be alleviated by rhein through the AMPK/mammalian target of rapamycin (mTOR) signaling pathway (Tu et al., 2017). Paeoniflorin could inhibit autophagy partly by inhibiting the RAGE/mTOR/autophagy pathway against AGE-induced mesangial cell dysfunction (Chen et al., 2017). Panax notoginsenoside has been shown to have a protective effect on cisplatin-induced kidney injury, mainly because of its ability to enhance the mitochondrial autophagy of renal tissue via the hypoxia inducible factor-1α/Bcl-2 adenovirus E1B 19 kDa interacting protein 3 pathway (Liu et al., 2015b). Treated with ginsenoside Rg1 could reduce aldosterone-induced autophagy in kidney epithelial cells, possibly through inhibiting the AMPK/mTOR pathway (Wang et al., 2015c).

### Antifibrotic Effect of Chinese Herbal Medicines

Renal fibrosis is defined by excessive deposition of extracellular matrix (ECM), which disrupts and replaces the functional parenchyma and leads to organ failure (Djudjaj and Boor, 2019). CKD and renal fibrosis affect half of adults older than 70 years of age and 10% of the world's population (Humphreys, 2018). Renal fibrosis is the final pathological process common to all forms of CKD. Targeting the TGF-β/Smads signaling pathway could be an effective strategy for the treatment of kidney diseases, because inhibition of the TGF-β/Smads signaling pathway ameliorates renal fibrosis and renal injury (Sun et al., 2016).

Astragaloside could attenuate the progression of renal fibrosis by suppressing fibroblast proliferation, transdifferentiation, and ECM production *in vivo* and vitro (Xu et al., 2014a; Che et al., 2015; Wang et al., 2015d; Chen et al., 2019b). *Salvia miltiorrhiza* Bunge could inhibit renal fibrosis induced by HgCl_2_, streptozotocin, and 5/6 nephrectomy, as well as HK-2 cells triggered by TGF-β1, which were related to the regulation of the TGF-β1/Smads pathway (Wang et al., 2010; Lee et al., 2011; Pan et al., 2011; Wang et al., 2015b). Triptolide attenuated tubulointerstitial fibrosis in rats with UUO, and its effect on renal fibrosis was similar to that of mycophenolate mofetil (Yuan et al., 2011). Tripterygium glycoside and triptolide could alleviate renal fibrosis involving the miR-141-3p/phosphatase and tensin homolog/AKT/mTOR pathway, TGF-β1/Smad3 pathway and TLR4/NF-κB pathway (Cao et al., 2015; Ma et al., 2015b; Li et al., 2017c). *Abelmoschus manihot* (L.) Medik. and the flavonoids components prevent tubulointerstitial fibrosis in chronic renal failure rat via inhibiting the NADPH oxidase/ROS/ERK pathway (Cai et al., 2017). *Abelmoschus manihot* (L.) Medik. also showed a renoprotective effect via attenuating renal fibrosis in a diabetic nephropathy rat model (Mao et al., 2015; Yang et al., 2019a). Puerarin could ameliorate renal fibrosis by inhibiting oxidative stress induced-epithelial cell apoptosis through MAPK signaling (Zhou et al., 2017a). Treatment of diabetic nephropathy rats with puerarin could increase the activity of matrix metalloproteinase-9, consequently degrading the ECM accumulated in the kidney (Tripathi et al., 2017). *Rheum palmatum* L. could ameliorate renal damage induced by UUO and immunoglobulin A nephropathy via reversing abnormal serum and urine biochemical parameters, as well as decreasing the production of fibrotic markers including fibronectin, collagen I, collagen III, and *α*-smooth muscle actin (Chen et al., 2015b; Chen et al., 2019d; Dou et al., 2020). Notoginsenoside R1 may be beneficial for ameliorating apoptosis and renal fibrosis induced by oxidative stress (Zhang et al., 2019a). Ginsenoside Rg1 has an antifibrotic effect by targeting Klotho/TGF-β1/Smad signaling in rats with UUO (Li et al., 2018).

The above research directions are not independent of each other. CHMs may play a renoprotective role through various ways, rather than a single target. There are also some other CHMs, including *Reynoutria japonica Houtt., Paeonia × suffruticosa* Andrews, *Epimedium brevicornu* Maxim., *Coptis chinensis* Franch., *Rehmannia glutinosa* (Gaertn.) DC., *Lycium barbarum* L., and *Bupleurum chinense* DC., that are used by practitioners of CHMs without clinical studies (Table 1). *In vivo* and vitro, these CHMs have been observed to have renoprotective effects based on their anti-inflammatory, antioxidative, anti-apoptotic, autophagy-mediated, and antifibrotic effects.

**TABLE 1:** Summary of the mechanism of commonly used CHMs.

Herbal Name	Main Active Compounds	Anti-inflammatory	Antioxidative	Anti-apoptotic	Autophagy- mediated	Antifibrotic	Indications
*Astragalus mongholicus* Bunge	Astragaloside (Gao et al., 2012; Gui et al., 2013; Wang et al., 2014; Xu et al., 2014a; Che et al., 2015; Wang et al., 2015d; Guo et al., 2017; Zhou et al., 2017b; Chen et al., 2018; Du et al., 2018; Ji et al., 2018; Chen et al., 2019b; Ju et al., 2019; Qu et al., 2019; Su et al., 2019; Wang et al., 2019c), calycosin (Tang et al., 2011; Zhang et al., 2019f)	+++	+++	+++	+++	+++	CKD Zhang et al. (2014a), DN Li et al. (2011), nephrotic syndrome Feng et al. (2013)
*Salvia miltiorrhiza* Bunge Lee et al. (2011); Cao et al. (2017)	Magnesium lithospermate B (Park et al., 2017; Lin et al., 2019a; Wang et al., 2019b; Zhao et al., 2019), salvianolic acid B (Wang et al., 2010; Pan et al., 2011; Ma et al., 2017), salvianolic acid A (Hou et al., 2017), tanshinone IIA (Liu et al., 2011; Wu et al., 2012b; Wang et al., 2015b; Jiang et al., 2016)	+++	+++	+	++	+++	DN Shen et al. (2020), hypertensive nephropathy Xu et al. (2019b)
*Tripterygium wilfordii Hook. f.*	Tripterygium glycoside (Wan et al., 2010; Cheng et al., 2015a; Ma et al., 2015b; Wu et al., 2017; Gong et al., 2018; Zhan et al., 2019; Wang et al., 2020), triptolide (Gao et al., 2010; Yuan et al., 2011; Ma et al., 2013; Cao et al., 2015; Zhou et al., 2016; Dong et al., 2017; Li et al., 2017c; Liang et al., 2018; Yang et al., 2019b; Wan et al., 2020), celastrol (Yu et al., 2018; Zhang et al., 2019c)	+++	++	+++	+++	+++	CKD Wang et al. (2018), DN Ren et al. (2019), primary nephrotic syndrome Chen et al. (2013c)
*Abelmoschus manihot* (L.) Medik. Zhou et al. (2012); Tu et al. (2013); Mao et al. (2015); Ge et al. (2016); Cai et al. (2017); Liu et al. (2017); Han et al. (2019); Li et al. (2019); Yang et al. (2019a)	Hyperoside (Wu et al., 2019)	+++	+++	++		++	DN Shi et al. (2019), primary glomerular disease Zhang et al. (2014b), IgA nephropathy Li et al. (2020a)
*Pueraria montana* (Lour.) Merr.	Puerarin (Kim et al., 2010; Zhong et al., 2014; Pan et al., 2015; Song et al., 2016; Zhou et al., 2017a; Li et al., 2017b; Song et al., 2017; Tripathi et al., 2017; Wang et al., 2019a; Li et al., 2020b; Liu et al., 2020)	++	+++	++	++	++	Stage III DN Wang et al. (2015a)
*Rheum palmatum* L. Zeng et al. (2013)	Rhein (Chen et al., 2015b; Meng et al., 2015; Tu et al., 2017; Bi et al., 2018; Chen et al., 2019d; Hu et al., 2020; Wu et al., 2020b), chrysophanol (Dou et al., 2020)	++	++	++	++	+++	CKD Wang et al. (2012a)
*Paeonia lactiflora* Pall.	Paeoniflorin (Chen et al., 2017; Lu et al., 2017; Zhang et al., 2017b; Liu et al., 2019a; Shao et al., 2019), total glucosides of paeony (Su et al., 2010; Xu et al., 2014b)	+++	+	+	+		DN Zhu et al. (2016)
*Panax notoginseng* (Burkill) F.H.Chen	Notoginsenoside (Liu et al., 2010; Liu et al., 2014; Liu et al., 2015b; Du et al., 2016b; Liu et al., 2019b; Zhang et al., 2019a; Zhang et al., 2019e; Fan et al., 2020)	++	+++	+++	+	+	DN Tang et al. (2020)
*Panax ginseng* C.A.Mey. Caliskan et al. (2015)	Ginsenoside Rg1 (Wang et al., 2015c; Ni et al., 2017a; Du et al., 2018; Li et al., 2018), Rb1 (Gao et al., 2020), Rh2b (Qi et al., 2019), ginseng total saponin (Ha and Ha, 2019).	++	++	++	+	+	Early CKD Xu et al. (2017)
*Reynoutria japonica* Houtt.	Polydatin (Xie et al., 2012; Chen et al., 2013a; Gao et al., 2015; Liu et al., 2015a; Sohn et al., 2015; Chen et al., 2016a; Ni et al., 2017b; Gu et al., 2019)	+++	+++	++		++	CKD ^b^
*Paeonia × suffruticosa* Andrews Zhang et al. (2014c); Zhang et al. (2014d)	Paeono (Liu et al., 2018; Zhang et al., 2018), terpene glycoside component from *Paeonia × suffruticosa* Andrews (Chen et al., 2016b)	+++	++			++	CKD ^b^
*Epimedium brevicornu* Maxim.	Icariin (Qi et al., 2011; Li et al., 2013; Liang et al., 2014; Huang et al., 2015; Ma et al., 2015a; Zhang et al., 2017a; Qiao et al., 2018; Su et al., 2018; Xie et al., 2018; Chen et al., 2019a)	+++	++	++		++	CKD ^b^
*Coptis chinensis* Franch. Ren et al. (2018)	Berberine (Liu et al., 2008a; Liu et al., 2008b; Liu et al., 2009; Wu et al., 2012a; Li and Zhang, 2017; Zhang et al., 2019d)	++	++	+		++	CKD ^b^
*Rehmannia glutinosa* (Gaertn.) DC.	Catalpol (Dong and Chen, 2013; Zhu et al., 2015; Zhang et al., 2019b; Chen et al., 2019c; Chen et al., 2020)	++	++	++		+	CKD ^b^
*Lycium barbarum* L.	Lycium barbarum polysaccharides (Du et al., 2016a; Liao et al., 2019; Lu et al., 2019; Wu et al., 2020a)	++	+			+	CKD ^b^
*Bupleurum chinense* DC.	Bupleurum polysaccharides (Liu et al., 2019c), saikosaponin B2 (Ren et al., 2020)	+				++	CKD ^b^

Abbreviations: CKD, chronic kidney disease; DN, diabetic nephropathy. Mechanisms were confirmed by multiple *in vitro* and *in vivo* studies, +++; mechanisms were shown only in two to four studies, ++; mechanism was shown only in one study, +. ^b^ Used by traditional medicines practitioners without clinical studies.

## Clinical Studies of Chinese Herbal Medicines in Treating Kidney Diseases

In this section, we will review the large and well-designed randomized controlled trials (RCTs) in peer-reviewed English-language journals. Although most studies on the treatment of CKD with CHMs were published in Chinese journals, systematic reviews and meta-analyses including these clinical trials were discussed. However, the methodological quality of the meta-analyses is generally low, with significant heterogeneity and publication bias.

### Randomized Controlled Trials of Chinese Herbal Medicines in Treating Kidney Diseases

To explore the therapeutic effect of Tangshen Formula in patients with diabetic nephropathy, 180 patients with deficiency of both Qi and Yin with blood stasis syndrome were randomly assigned to receive Tangshen Formula or a placebo based on conventional treatment (Li et al., 2015) (Table 2). After 24 weeks of treatment, there was no difference in the change of urinary albumin excretion rate (UAER) between the Tangshen Formula group and placebo group (*p* = 0.70). Compared with the placebo, Tangshen Formula significantly decreased 24 h urinary protein (24 h-UP) (*p* = 0.03). The estimated glomerular filtration rate (eGFR) was improved in both patients with microalbuminuria and macroalbuminuria. There was no significant difference in the proportion of adverse events between the Tangshen Formula group and placebo group. Tangshen Formula seems to provide additional benefits in reducing proteinuria and improving eGFR in diabetic nephropathy patients with macroalbuminuria. However, a comprehensive assessment of efficacy and safety of Tangshen Formula requires long-term follow-up and hard end points, such as doubling of baseline serum creatinine (SCr), ESRD, and death. In order to evaluate the renoprotective effect and safety of Tangshen Formula fully, it is necessary to conduct a long-term follow-up.

**TABLE 2:** Large and well-designed RCTs with CHMs.

Study	N	Therapeutic Arms	Disease	Primary Outcomes	Duration of Intervention (weeks)	Outcomes
Li et al. (2015)	180	TSF vs. placebo	Diabetic nephropathy	Changes of UAER and 24 h-UP	24	UAER: −19.53 (−52.47, 13.41) vs. −7.01 (−47.33, 33.73) μg/min; *p* = 0.70. 24 h UP: −0.21 (−0.48, 0.06) vs. 0.36 (−0.04, 0.76) g/24 h; *p* = 0.03
Zhang et al. (2014b)	417	*Abelmoschus manihot* (L.) Medik. vs. losartan vs. *Abelmoschus manihot* (L.) Medik. + losartan	Primary glomerular disease	Change in 24 h-UP	24	-508 ± 457 vs. −376 ± 577 (*p* = 0.003) vs. −545 ± 500 mg/24 h (*p* < 0.001)
Qiu et al. (2014)	479	Rehmannia glutinosa acteosides + irbesartan vs. irbesartan	Primary chronic glomerulonephritis	Percent change of 24 h-UP	8	36.42 ± 43.17 vs. 27.97 ± 50.28%; *p* = 0.03
Chen et al. (2013b)	190	Shenqi particle vs. prednisone + cyclophosphamide	Idiopathic membranous nephropathy	Complete remission or partial remission	48	46/63 (73.0%) vs. 54/69 (78.3%); *p* = 0.5
Wang et al. (2012b)	578	CHMs vs. benazepril vs. CHMs vs. CHMs + benazepril	Primary glomerulonephritis in CKD stage 3	eGFR	24	48.46 ± 15.90 vs. 43.00 ± 12.37 vs. 48.31 ± 17.50 ml/min; *p* < 0.05

TSF, Tangshen Formula; UAER, Changes of urinary albumin excretion rate; 24 h-UP, 24-h urinary protein; CHMs, Chinese herbal medicines; eGFR, estimated glomerular filtration rate.

Another study recruited 417 patients with primary glomerular disease in a 26-center, randomized, controlled, open-label, clinical trial (Zhang et al., 2014b) (Table 2). Patients were randomly assigned to receive *Abelmoschus manihot* (L.) Medik., losartan, or *Abelmoschus manihot* (L.) Medik. combined with losartan. After 24 weeks of treatment, *Abelmoschus manihot* (L.) Medik. was more effective in reducing proteinuria than losartan (50 mg/day). In addition, *Abelmoschus manihot* (L.) Medik. combined with losartan was more effective than losartan alone. There was no significant difference in the mean eGFR and adverse events between the three groups. Some limitations of the study were a lack of patients with nephrotic syndrome, the exclusion of secondary glomerular diseases, and short follow-up time. *Abelmoschus manihot* (L.) Medik. may be an alternative treatment for primary kidney disease with moderate proteinuria and fairly maintained kidney function (eGFR ≥60 ml/min/1.73 m^2^).

One study recruited 479 patients with primary chronic glomerulonephritis (Qiu et al., 2014) (Table 2). Patients were randomly divided into a treatment group (Rehmannia glutinosa acteosides combined with irbesartan) and a control group (irbesartan). After 8 weeks of treatment, the treatment group showed a reduction in 24 h-UP compared to baseline, which was significantly higher than that in the control group (*p* = 0.03). The proportion of adverse events was similar between the two groups. Rehmannia glutinosa acteosides combined with irbesartan was more effective in reducing proteinuria in patients with chronic glomerulonephritis than irbesartan alone. Because most of the included patients had mild, chronic lesions, future trials should confirm whether Rehmannia glutinosa acteosides combined with irbesartan can be effective in patients with complex kidney diseases.

In an open-label, parallel, randomized, controlled clinical trial, 190 patients with idiopathic membranous nephropathy were recruited from seven hospitals (Chen et al., 2013b) (Table 2). All patients had nephrotic syndrome and an eGFR level >30 ml/min/1.73 m^2^. The difference in change of proteinuria was not significantly different between the two groups (*p* = 0.6). Patients receiving Shenqi particle showed significant improvement in eGFR levels compared with the control group (*p* = 0.005). Severe adverse events mainly occurred in the control group. Shenqi particle may be a potential complementary therapy for patients with idiopathic membranous nephropathy and nephrotic syndrome. In this study, patients were not observed for 3–6 months in order to exclude those with spontaneous remission. This study was also limited by a high dropout rate and lack of a prior observation period.

Another study recruited 578 patients with primary glomerulonephritis in CKD stage 3 (Wang et al., 2012b) (Table 2). Patients were randomly assigned to a CHMs group, benazepril group, or CHMs combined with benazepril group for 24 weeks. The CHMs used in this study were designed according to four different TCM syndromes: replenishing qi and blood decoction for the treatment of Qi Yin/Xue deficiency patterns, promoting blood flow decoction for the treatment of blood stasis in the kidney patterns, expel wind-evil and remove wetness decoction for the treatment of wind-dampness interfering in the kidney patterns, and clearing heat and dissipating dampness decoction for the treatment of patterns of endoretention of damp heat. Compared with baseline, eGFR levels in the CHMs group and the CHMs combined with benazepril group were improved, while there was no change in the benazepril group. Compared with the CHMs group, 24 h-UP and urinary albumin/creatinine levels decreased in the combined group and benazepril group (*p* < 0.05). Dry cough was most common in the combined group and benazepril group (*p* < 0.05). As a result, CHMs combined with benazepril ameliorated renal function and decrease proteinuria synergistically.

### Systematic Reviews and Meta-Analyses of Chinese Herbal Medicines in Treating Kidney Diseases

A systematic review and meta-analysis was performed on RCTs and quasi-RCTs comparing *Astragalus mongholicus* Bunge used alone with a placebo, no treatment, or conventional interventions in patients with CKD (Zhang et al., 2014a). The study included 22 trials with 1,323 participants, and showed that *Astragalus mongholicus* Bunge significantly increased the creatinine clearance (CrCl) and decreased SCr, especially in those with a baseline SCr level <133 μmol/L. *Astragalus mongholicus* Bunge also decreased 24 h-UP and BP, as well as increased hemoglobin and serum albumin. Six of 22 of the included studies reported no adverse effects, while the remaining studies did not report adverse effects. In six trials, the risk of bias was assessed as high, whereas it was not clear in the remaining 16 trials. The quality of the included studies was low overall. Although *Astragalus mongholicus* Bunge combined with conventional therapy has some promising effects in reducing proteinuria and increasing hemoglobin and serum albumin, the reliability of the results is affected by poor methodological quality and insufficient reports. Other meta-analyses showed that *Astragalus mongholicus* Bunge may have a renal protective effect on diabetic nephropathy (Li et al., 2011) and nephrotic syndrome (Feng et al., 2013).

Reviewing RCTs of diabetic nephropathy, a meta-analysis included 12 studies with 1,030 patients, and showed that the combination of salvianolate and Western medicine had renoprotective, anti-inflammatory, and antioxidative effects by reducing levels of SCr, blood urea nitrogen (BUN), urine protein, hypersensitive C-reactive protein (CRP), interleukin-6, MDA, as well as increasing the level of SOD. Compared with single-use Western medicine, the combination did not increase the occurrence of serious adverse events (Shen et al., 2020). Salvianolate can be considered as a promising alternative therapy for diabetic nephropathy. To evaluate the efficacy and safety of a sodium tanshinone IIA sulfonate injection, the extractive of *Salvia miltiorrhiza* Bunge, in the treatment of hypertensive nephropathy, a systematic review and meta-analysis included 16 trials comprising 1,696 participants, and indicated that sodium tanshinone IIA sulfonate in combination with an ARB was more effective than ARB monotherapy in improving the eGFR level and reducing 24 h-UP, SCr, cystatin-C, urinary immunoglobulin G, and urinary transferrin levels. In addition, the combination therapy controlled BP better than the monotherapy, and no adverse drug reactions were observed (Xu et al., 2019b). The effect of sodium tanshinone IIA sulfonate injection in addition to ARBs on the renal function of patients with primary hypertensive nephropathy is stronger than that of ARB alone. The combination therapy provides an auxiliary antihypertensive effect; however, because of the low methodological quality and small sample sizes, more rigorously designed RCTs are needed to verify the renoprotective effect of *Salvia miltiorrhiza* Bunge extract.

A systematic review and meta-analysis review included 103 RCTs comparing the efficacy and safety of *Tripterygium wilfordii* Hook. f. with a placebo, conventional Western medicine and other immunosuppressive medicine in CKD. *Tripterygium wilfordii* Hook. f. has nephroprotective effects by decreasing 24 h-UP, SCr, and BUN levels, and decreasing the incidence of adverse reactions (Wang et al., 2018). Although *Tripterygium wilfordii* Hook. f. combined with an ACEI/ARB increased the risk of adverse events, the combination in the treatment of diabetic nephropathy stage IV was superior to the monotherapy of ACEI/ARB (Ren et al., 2019). *Tripterygium wilfordii* Hook. f. may have an add-on effect on remission in patients with primary nephrotic syndrome (Chen et al., 2013c).

A systematic review and meta-analysis review included 72 RCTs to assess efficacy and safety of *Abelmoschus manihot* (L.) Medik. in diabetic nephropathy (Shi et al., 2019). Compared to an ACEI/ARB, *Abelmoschus manihot* (L.) Medik. combined with an ACEI/ARB was more effective on 24 h-UP, UAER, and 24 h-UP reduction rate values and normalization of UAER and SCr values; further, *Abelmoschus manihot* (L.) Medik. did not increase the risks of adverse events. Thus, *Abelmoschus manihot* (L.) Medik. combined with an ACEI/ARB can effectively and safely reduce proteinuria and protect renal function in patients with diabetic nephropathy.

Reviewing RCTs of diabetic nephropathy, a meta-analysis included 10 studies with 669 patients; the authors showed that puerarin combined with an ACEI significantly decreased the UAER, but it had no effect on 24 h-UP, BUN, and SCr levels. One trial reported abdominal discomfort and nausea (two cases) in the treatment group (Wang et al., 2015a). Puerarin may be considered as a beneficial therapy for diabetic nephropathy.

A systematic review and meta-analysis review included nine RCTs and quasi-RCTs to assess the efficacy and safety of *Rheum palmatum* L. in CKD (Wang et al., 2012a). Compared with no treatment, *Rheum palmatum* L. had a positive effect on SCr and BUN. Compared with captopril, *Rheum palmatum* L. had no significant effect on the BUN level, CrCl level, or patients’ capacity to undertake work. Only minor adverse events were reported with *Rheum palmatum* L. At present, the effective evidence of *Rheum palmatum* L. in improving SCr and BUN levels in patients with CKD is scarce and of low quality. Although *Rheum palmatum* L. does not seem to be associated with serious adverse events, there is no evidence to support its use in patients with CKD.

Another systematic review and meta-analysis review included 24 RCTs to assess efficacy and safety of *Panax notoginseng* (Burkill) F.H.Chen in diabetic nephropathy (Tang et al., 2020). Compared with conventional medicines, *Panax notoginseng* (Burkill) F.H.Chen combined with conventional medicines was associated with reductions of albuminuria, proteinuria, and levels of SCr, total cholesterol, triglycerides, and low-density lipoprotein cholesterol. However, none of the included trials mentioned adverse events. *Panax notoginseng* (Burkill) F.H.Chen can significantly improve renal function and lipid metabolism in diabetic nephropathy.

## Discussion

In recent years, with the further research studies on the efficacy and mechanism of TCM in the treatment of CKD, Chinese medicine may play an important role in relieving proteinuria and delaying ESRD. Compared with chemical agents targeting single molecular targets, CHMs containing different ingredients have advantages in the treatment of CKD. However, there are some concerning issues. Firstly, the clinical application of TCM is mostly a mixed formulation with unknown active ingredients, which is complex and difficult to analyze. Secondly, because of the generally low methodological quality, significant heterogeneity, and publication bias of meta-analyses, high-quality RCTs are required to confirm these findings before the routine use of CHMs. Thirdly, attention should be paid to the safety of CHMs. For example, astragaloside IV can cause growth retardation, so pregnant women should use it with caution (Li et al., 2017a); adverse reactions induced by *Tripterygium wilfordii* Hook. f. are systemic and organ-specific, which is related to the processing of medication, combined intervention, and drug dosage (Ru et al., 2019); and elderly subjects are vulnerable to the toxicity of a high dosage of *Rheum palmatum* L., which prompted people to consider the rational use of *Rheum palmatum* L. in the elderly population (Wang et al., 2011).

According to the published protocol, several well-designed prospective RCTs are currently ongoing to study CHMs in patients with CKD. In the future, we need to further identify the mechanisms and active ingredients of CHMs by modern technologies, including bioinformatics, network pharmacology, and high-throughput mass spectrometry.

## Conclusions

Recent studies on the mechanisms of kidney disease *in vitro* and in animal models have shown that CHMs have anti-inflammatory, antioxidative, anti-apoptotic, autophagy-mediated, and antifibrotic effects. Several well-designed RCTs and meta-analyses demonstrated that CHMs as an adjuvant to conventional medicines may benefit patients with CKD. CHMs is a potential method in the treatment of CKD. Further study on the mechanism and well-conducted RCTs are urgently needed to evaluate the efficacy and safety of CHMs.
